# Current Bioengineering Methods for Whole Kidney Regeneration

**DOI:** 10.1155/2015/724047

**Published:** 2015-05-18

**Authors:** Shuichiro Yamanaka, Takashi Yokoo

**Affiliations:** Division of Nephrology and Hypertension, Department of Internal Medicine, Jikei University School of Medicine, Tokyo 105-8461, Japan

## Abstract

Kidney regeneration is likely to provide an inexhaustible source of tissues and organs for immunosuppression-free transplantation. It is currently garnering considerable attention and might replace kidney dialysis as the ultimate therapeutic strategy for renal failure. However, anatomical complications make kidney regeneration difficult. Here, we review recent advances in the field of kidney regeneration, including (i) the directed differentiation of induced pluripotent stem cells/embryonic stem cells into kidney cells; (ii) blastocyst decomplementation; (iii) use of a decellularized cadaveric scaffold; (iv) embryonic organ transplantation; and (v) use of a nephrogenic niche for growing xenoembryos for *de novo* kidney regeneration from stem cells. All these approaches represent potentially promising therapeutic strategies for the treatment of patients with chronic kidney disease. Although many obstacles to kidney regeneration remain, we hope that innovative strategies and reliable research will ultimately allow the restoration of renal function in patients with end-stage kidney disease.

## 1. Introduction

Transplantation represents the ideal method of restoring organ function in patients with organ failure. However, the lack of suitable available donor organs is a serious problem worldwide [1]. Regenerative medicine has the potential to provide the ultimate treatment for various diseases by using autologous cells to reconstruct new organs and replace failing organs, and it has thus garnered considerable attention in recent years.

**Figure 1:**
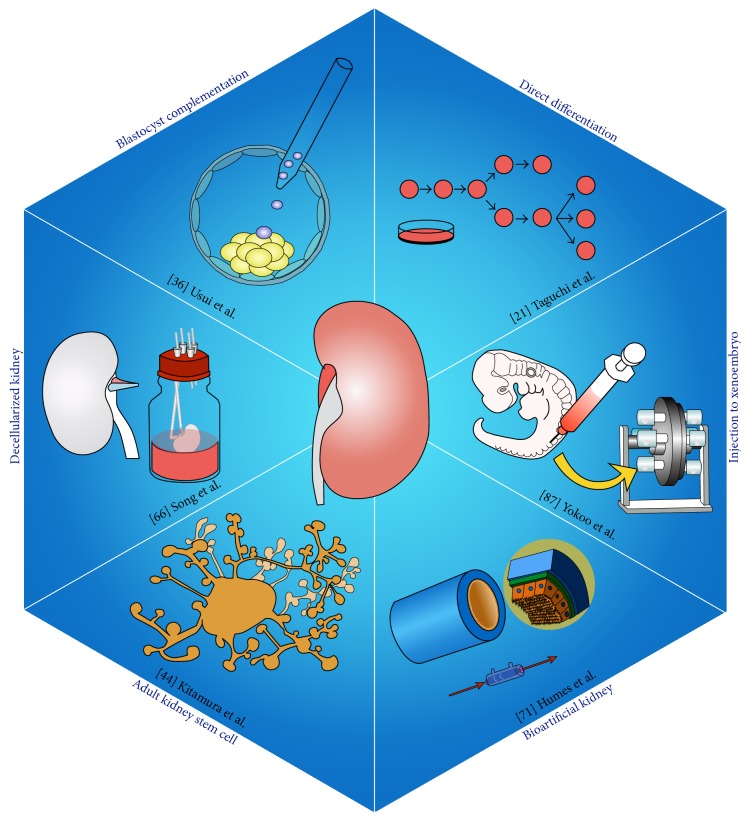
Schematic representation of the main strategies for kidney regeneration.

There are currently no published clinical reports regarding the regeneration of functional organs for the treatment for terminal organ failure. Kidney regeneration is particularly difficult because of the anatomical complexity of the organ [2], and reconstruction of the kidney's cubic structure is hard. The kidneys function to maintain homeostasis and are responsible for blood filtration and urine production, as well as the control of endocrine functions via erythropoietin (Epo) and vitamin D. To date, regeneration of all the constituent cells of the kidney has not been achieved. However, it is possible that kidney regeneration may be achieved by gradual advances in stem cell research and cellular engineering (Figure 1).

The regeneration of an entire kidney may be difficult, but functional recovery of as little as 10% of kidney filtration function might allow for the withdrawal of dialysis in patients with end-stage kidney disease (ESKD) [3], thus greatly increasing quality of life. The regeneration of a functional kidney represents a huge practical challenge. However, research in the field of regeneration is continuing to make progress, with the aim of overcoming ESKD through kidney regeneration. In this review, we consider current advances in kidney regeneration, including induced pluripotent stem cell (iPSC) technologies and renal bioengineering, and we discuss some of the associated limitations and challenges.

## 2. Directed Differentiation of Induced Pluripotent Stem Cells and Embryonic Stem Cells into Kidney Cells

Previous studies have suggested that pluripotent stem cells (PSCs) have the potential to differentiate into any cell type in the body and self-assemble into heterogeneous tissues or organs [4–6]. PSCs have indeed been shown to generate mature cells* in vitro*. PSCs therefore represent an important cell type for bioengineering strategies aimed at kidney regeneration. PSCs include both embryonic stem cells (ESCs) and iPSCs. ESCs are derived from embryos and grown in primary culture [4], while iPSCs are produced from terminally differentiated cells using transfection factors such as c-myc, Oct4, Klf4, and Sox-2 [5, 6]. PSCs have been differentiated successfully into various types of cells and tissues, including intestine [7], hepatic [8, 9], neural [10, 11], hematologic [12], pancreatic [13, 14], and cardiac lineages [15]. This approach, whereby iPSCs or ESCs are differentiated into kidney or other progenitors, is termed “directed differentiation” and is accomplished by the sequential application of chemicals or growth factors.

Recent progress in stem cell research has generated human nephron progenitor cells, including intermediate mesoderm (IM) and metanephric mesenchyme (MM) cells [16–21]. An understanding of the processes and molecular mechanisms of kidney development is important for the production of appropriate cells to generate nephrons. The kidney is derived from IM and arises from the ureteric bud and MM following precisely timed interactions between multiple signals [22]. Different studies have used different growth-factor protocols in human PSCs (hPSCs); however, the Wnt agonist CHIR99021 is commonly used to promote mesoderm differentiation [16].

High-throughput chemical screening and low-molecular-weight chemical compounds have recently been used in directed differentiation for kidney formation. An efficient method has been developed for inducing the differentiation of IM cells from hiPSCs/hESCs using a combination of activin A and CHIR99021 to generate mesoderm, followed by combined treatment with bone morphogenetic protein 7 (BMP7) and CHIR99021 [17, 18]. This protocol induced hiPSCs to develop into odd-skipped-related 1 (OSR1)+ IM cells with an efficiency greater than 90% [18]. Furthermore, high-throughput screening of approximately 1,821 chemical compounds identified two retinoic acid receptor agonists, AM580 and TTNPB, that efficiently induced the differentiation of human iPSCs (hiPSCs)/ESCs into IM cells [23]. The high-throughput screening system used a hiPSC reporter line, in which the green fluorescent protein (GFP) coding sequence was knocked into the OSR1 gene locus, allowing small molecules that increased the induction rate of OSR1+ cells to be identified quantitatively by flow cytometry. GFP expression was examined using an LSR Fortessa cell analyzer equipped with a high-throughput sampler (BD Biosciences, San Jose, CA, USA). They proposed that small chemical compounds were less expensive and more consistent than growth factors and might therefore be more suitable for generating IM cells [18, 23]. This study demonstrated the feasibility of monitoring the nephrogenic-differentiation capacity of hiPSCs and provided a new strategy for investigating the efficiency and specificity of methods of achieving renal differentiation of hiPSCs.

Many studies have attempted to generate MM progenitor cells by direct differentiation from PSCs. Xia et al. tried to differentiate PSCs into ureteric bud (UB) lineage cells by stepwise treatment of iPSCs/hESCs with a combination of fibroblast growth factor 2 (FGF2) and BMP4 for 2 days, followed by combined treatment with activin A and BMP2 for another 2 days. After the 4 days, IM-like cells expressing PAX2, OSR1, WT1, and LHX1 were produced. The UB markers HOXB7, RET, and GFRA1 were upregulated in these cells after an additional 2 days of differentiation, implying that PSCs could be driven towards UB progenitor-like cells. Additionally,* in vitro* differentiation of PSCs generated cells with a UB-committed IM fate with the potential to assemble spontaneously into complex, chimeric three-dimensional (3D) structures upon coculture with murine embryonic kidney cells [19]. However, the differentiation efficiency was poor, though the reasons for this were unclear.

The strategy of direct differentiation from PSCs has made significant advances in the past few years [20, 21]. Takasato et al. [20] and Taguchi et al. [21] noted that nephron progenitor cells were derived from PSCs. The developmental origin of the kidney is well known. Takasato et al. [20] induced a primitive streak from hESCs using activin A (or CHIR99021) and BMP4. The protocol up to this point was similar in both groups [20, 21], but the subsequent protocols used to induce IM differentiation from posterior primitive streak differed. The differentiated hESCs formed renal vesicles combined with dissociated embryonic mouse kidney cells in the study by Takasato et al., involving the integration of human cells into mouse renal structures [20]. In contrast, Taguchi et al. demonstrated more reasonable protocols for inducing renal structures such as nephrons and proximal tubules [21]. They proposed the concept of “posteriorization” for inducing nephron progenitors from PSCs in a mouse and human model [21]. Briefly, nephron progenitor cells of the MM could be derived from posteriorly located IM. Brachyury, encoded by the T gene, is a representative marker of the primitive streak and posterior nascent mesoderm [24]. Posteriorly located T+ mesoderm is a putative part of the axial progenitor cells recently identified as the source of the caudal body trunk [25]. They also assumed that there were differences in the developmental processes between the posteriorly located MM and anteriorly located mesodermal tissues such as the heart, which have been successfully induced from PSCs by way of the T+ state with an initial short period of differentiation [26]. The competence of the IM differed both temporally (E8.5 versus E9.5) and spatially (anteroposteriorly), and OSR1+ or PAX2+ cells were considered to represent a homogeneous population [21]. This study indicated that a precursor of the UB was located anteriorly in IM cells lacking T at E8.5 and was already segregated from that of the MM localized in the T+ posterior nascent mesoderm. Surprisingly, the T+ posterior nascent mesoderm was negative for OSR1 but nevertheless contained nephron progenitors [21]. Lineage-tracing experiments demonstrated that the MM precursor could be traced back to the OSR1+ posterior IM at E9.5 and T+/OSR1+ posterior mesoderm at E8.5 [21]. The same study also established an efficient protocol for inducing metanephric nephron progenitors from the posterior nascent mesoderm using a combination of high-CHIR99021 (10 *μ*M) and BMP4 for posteriorization, followed by combined treatment with mid-CHIR99021 (3 *μ*M) and BMP4 with the addition of retinoic acid and activin A. A final step involved the addition of low-CHIR99021 (1 *μ*M) and FGF9 for 1 day each. They finally verified the temporal kinetics of gene expression at each step of the induction process. Wnt signals are important for posteriorization [25, 27]. Indeed, a high concentration of CHIR (a Wnt agonist) was used in combination with BMP4 to maintain the posterior nascent mesoderm in the posteriorization phase [21]. They established the induction of MM from T+ caudal precursors. They also established stepwise protocols for the differentiation of both mouse ESCs and human iPSCs into metanephric nephron progenitors, thus enabling kidney generation using multiple stage-specific growth factors [21]. Coculture of embryoid bodies, containing nephron progenitors, with mouse embryonic spinal cord, a well-established inducer of kidney tubulogenesis, resulted in the formation of 3D tubular structures expressing markers characteristic of renal tubules and glomeruli [21]. Although the authors were unable to confirm urinary production or other kidney functions, this direct differentiation method for kidney regeneration appears to be the most complete and reliable study published to date.

Generating precise renal progenitor cells is essential for the development of a whole kidney* de novo*. The differentiation of induced IM cells into precise renal progenitor cells would allow a complete 3D kidney structure to be constructed from PSCs. However, the means of successfully regenerating a functional vascular system between the regenerated kidney and the recipient remain unknown. Additionally, the* in vivo* functioning of a regenerated kidney remains unclear. However, further advances in developmental biology and bioengineering may resolve these issues and allow whole kidney regeneration.

## 3. Blastocyst Complementation

Injection of PSCs into blastocysts, the initial embryonic stage after fertilization, synchronizes the development of two line cells, and the combined blastocyst generates a chimeric body. In the first report of this method, normal ESCs were injected into blastocysts of recombination-activating gene 2-deficient mice, which have no mature B or T lymphocytes, to generate somatic chimeras with ESC-derived mature B and T cells [28]. This “blastocyst complementation” system was applied to the reconstruction of several tissues and organs, including thymic epithelium [29], heart [30], yolk sac hematopoiesis [31], germ cells [32], hepatocytes [33], pancreas [34, 35], and kidney [36]. A recent study to generate a functional organ using blastocyst complementation by Kobayashi et al. showed that the injection of rat iPSCs into Pdx1^−*/*−^ (pancreatogenesis-disabled) mouse blastocysts produced newborn rat/mouse chimeras with a pancreas derived almost entirely from rat iPSCs [34]. The mouse and rat PSC-derived pancreas produced insulin, and the transplantation of PSC-derived pancreas islets improved hyperglycemia in a diabetic rodent model [37]. This study indicated that PSC-derived cellular progeny could occupy and develop in a vacant developmental niche. Furthermore, these results also demonstrated that interspecific blastocyst complementation could be used to generate organs derived from donor PSCs* in vivo* using a xenogeneic environment [36, 37]. This blastocyst complementation system has already been applied to whole kidney reconstruction [36]. Nondeficient murine iPSCs were injected into blastocysts from kidney-deficient mice lacking the SAL-like 1 protein essential for kidney development, and the neonatal mice had kidneys derived almost entirely from injected iPSCs [36]. However, the vascular and nervous systems were not constructed from cells of iPSC origin, and the kidney was therefore not completely complemented. Immunohistochemical analysis of the regenerated kidney indicated that the renal vascular system, including renal segmental, lobar, interlobar, arcuate, and interlobular arterioles, was a chimeric structure originating from both host cells and donor iPSCs [36]. Precise urinary analysis was not carried out and whether or not filtrated and reabsorbed urine was produced is unclear. Moreover, injection of rat iPSCs into kidney-deficient mouse blastocysts failed to generate rat kidneys in mice. This result implies that the key molecules in mice involved in interactions between the mesenchyme and UB do not cross-react with those in rats. The generation of xenogeneic organs using interspecific blastocysts thus requires a host animal strain lacking all renal lineages [36].

The most important problem associated with blastocyst complementation is the ethical issue. It is impossible to exclude the possibility of generating interspecific chimeras containing brain derived from injected PSCs. Although it is difficult to establish a xenogeneic blastocyst complementation system that overcomes the xenogeneic barrier, this strategy appears to be one of the most promising methods for kidney regeneration.

## 4. Adult Kidney Stem/Progenitor Cell Reconstitution 

Stem/progenitor cells isolated from many adult organs demonstrate self-renewing ability and can give rise to terminally differentiated cells. This kidney stem/progenitor population disappears in the adult kidney, possibly because of the loss of its niche [37, 38]. However, renal stem/progenitor cells still exist in specific locations in the adult kidney, such as in the renal papilla [39], tubular epithelial cells [40], Bowman's capsule [41], and the S3 segment of the proximal tubules [42, 43]. Kitamura et al. recently reported that adult kidney stem/progenitor cells derived from the S3 segment of adult rat kidney nephrons were able to reconstitute a 3D kidney-like structure* in vitro* [44]. Kidney-like structures were formed when a cluster of kidney stem/progenitor cells was suspended in an extracellular matrix gel and cultured in the presence of several growth factors (combination of glial cell-derived neurotrophic factor, basic FGF, hepatocyte growth factor, epidermal growth factor, and BMP7). The clusters from dissociated S3-segment cells were induced by the hanging-drop method in 3D culture [44], while 2D culture conditions were unable to reconstruct kidney-like structures. Surprisingly, the reconstructed kidney-like structures included all the kidney substructures, including glomeruli, proximal tubules, the loop of Henle, distal tubules, and the collecting ducts, but not the vasculature. They suggested that a cluster of tissue stem/progenitor cells may have the ability to reconstitute the minimum unit of its organ of origin by differentiating into specialized cells in the correct niche. Kidney stem/progenitor cells derived from the S3 segment of adult rat kidney have been shown to express stem cell markers such as Sca-1, c-kit, nestin, and Musashi-1, together with renal lineage markers such as PAX-2 and WT-1 [44]. They assumed that these cells were similar to metanephric mesenchymal cells, based on marker protein expression. However, the clusters can differentiate into collecting-duct-like cells or mesangial-like cells, which are not thought to be derived from MM [44]. In this regard, the question of whether adult kidney stem cells can differentiate into lineages other than UB or MM remains to be answered. The kidney-like structures were not vascularized and did not produce urine. However, adult kidney stem cells remain poorly understood. The potential tumorigenicity of undifferentiated hiPSCs is a critical problem for these cells as a clinical source, while adult stem/progenitor cells have been reported to be nontumorigenic when injected into a mouse model [45–47]. These reports raise the possibility that adult stem cells may represent a safer clinical source than PSCs. If it is possible to establish adult stem/progenitors with multipotency for kidney reconstruction, they may be promising cellular source for kidney repair and regeneration.

## 5. Decellularized Cadaveric Scaffold

Native kidney extracellular matrix (ECM) has been reported to provide a scaffold for cell seeding and a niche for stem cells to differentiate into whole organs [48]. The ECM plays a crucial role in kidney development and repair [48–52]. ECM molecules and their receptors influence organogenesis and repair by providing a scaffold for the spatial organization of cells, by secreting and storing growth factors and cytokines, and by regulating signal transduction [48–53]. ECM scaffolds from whole human-cadaveric and animal organs can be generated by detergent-based decellularization [1, 54]. This strategy was used by Ott et al. [55] to develop a functional rat heart. A whole-heart scaffold with intact 3D geometry and vasculature was prepared by coronary perfusion with detergents into the cadaveric heart. The rat heart was then seeded with neonatal cardiac cells or rat aortic endothelial cells, which subsequently induced the formation of contractile myocardium that performed stroke work [55]. Decellularized cadaveric scaffolds have also been used in several other organ systems, including the liver [56], respiratory tract [57], nerves [58], tendons [59], valves [60], bladder [61], and mammary gland [62]. Furthermore, some studies have used decellularization-recellularization technology for kidney regeneration. Many animals have been used for decellularization studies, including rats [63], rhesus monkeys [64], and pigs [65]. However, regenerated kidneys produced by this method did not have sufficient renal function to produce urine and Epo. Song et al. recently described the successful regeneration of a whole kidney that produced urine after transplantation [66]. Notably, they generated 3D acellular renal scaffolds by perfusion decellularization of cadaveric rat, pig, and human kidneys. Endothelial and epithelial cells were repopulated by perfusion, leading to the formation of viable tissues for renal construction. However, the mechanism whereby the infused cells differentiate and are orchestrated into nephrons with vasculature to produce urine remains unclear. Decellularized cadaveric scaffolds are associated with the problem of massive thrombi, despite strong anticoagulation prophylaxis. Although this strategy still has many obstacles, it demonstrates the impact of regenerative medicine on organ transplantation and its potential as a solution for the shortage of donor organs.

## 6. Tissue Engineering of a Bioartificial Kidney: Renal Tubule Assist Device 

The developing field of tissue engineering is an extension of cell therapy, in which biological and engineering science techniques are combined to create structures and devices to replace lost tissue or organ functions [67, 68]. The development of bioartificial kidneys (BAKs) represents the intersection between regenerative medicine and renal replacement therapy [52]. A renal tubule assist device (RAD) containing living renal proximal tubule cells has been successfully engineered, and it demonstrated differentiated absorptive, metabolic, and endocrine functions similar to normal kidneys in animal experiments* in vitro* and* ex vivo* [69]. Briefly, renal proximal tubule segments were harvested from kidneys, and renal tubule progenitor cells were selected and expanded [70]. The tubule progenitor cells were grown in culture dishes with culture medium containing specific additives [68]. A RAD with high-flux hemofiltration cartridges containing polysulfone hollow fibers coated with pronectin-L was used as a scaffold device [68]. Renal proximal tubule cells were then seeded into the hollow fibers and the seeded cartridge was connected to a bioreactor perfusion system, in which the extracapillary space was filled with culture medium and the intracapillary space was perfused with medium. The cell cartridges were used at least 14 days after seeding. The RAD units included confluent monolayers of renal proximal tubule cells with characteristics including microvilli, tight junctional complexes, and endocytic vesicles demonstrated by transmission electron microscopy [68]. The tissue-engineered bioartificial RAD performed differential reabsorption and secretory transport because of the specific active transporters present in the proximal tubule cells* in vivo*. However, these transport functions were less efficient than those in native proximal tubules [68]. The same group reported that the RAD was able to maintain viability in a uremic environment in uremic dogs with acute renal failure when placed in series with a conventional hemofilter and an extracorporeal blood circuit [71]. Furthermore, they also performed clinical trials with BAKs [72–74]. The combination of regenerative medicine and bioengineering thus offers promise for the regeneration of whole kidneys.

## 7. Embryonic Organ Transplantation

We attempted to regenerate a functional, transplantable whole kidney able to produce urine and renal hormones, such as Epo, using a xenoembryo and human mesenchymal stem cells. The embryonic metanephros, which is the mammalian renal anlagen, is thought to represent a potential source for the regeneration of functional whole kidneys [75–83]. An embryonic metanephros transplanted into a host animal (rat) was able to obtain its blood supply from the host [75]. Indeed, the survival of anephric rats was prolonged on the basis of renal function provided by a single transplanted rat renal anlagen, the ureter of which was anastomosed to a host ureter [83]. Furthermore, the transplanted metanephros produced renal hormones including Epo and renin, which elevated the blood pressure of the host animal [77, 78]. Metanephroi from porcine embryos implanted either in the omentum of mice in which costimulation was blocked [79] or under the kidney capsules of immunodeficient mice [81] developed fully functional nephrons. The levels of urea nitrogen and creatinine were higher in cyst fluid produced by the transplanted metanephroi than in sera from the transplanted host animals [81], suggesting urine production. Metanephros transplantation was also shown to reduce vascular calcification in rats with chronic renal failure [79] and to maintain blood pressure in anephric rats with induced acute hypotension [78], implying that the transplanted metanephroi carried out multiple renal functions, other than urine production. These results suggest that metanephros transplantation might be used to overcome the shortage of donor kidneys available for transplantation.

We recently demonstrated that xenotransplanted metanephros could supply endogenous MSCs with a niche for differentiation into Epo-producing tissues [84]. Polymerase chain reaction using species-specific primers and sequence analysis revealed that xenotransplanted metanephroi, either from rat to mouse or from pig to cat, expressed Epo of host animal origin. This indicated that the Epo-producing cells originated in the host animal and developed to produce Epo in the transplanted metanephros. We further showed that the Epo-producing cells did not originate from integrating vessels, but rather from circulating host MSCs mobilized from the bone marrow. Of note, conventional metanephros transplantation requires continuous and strong immunosuppression to avoid humoral rejection associated with the xenogeneic barrier, which can induce adverse effects including carcinogenicity and severe rejection. For safety purposes, the xenotransplant should thus be discarded when it is no longer required, by introducing a cell-fate-regulating system including a suicide gene that can be expressed on demand. To avoid the xenogeneic barrier, we used metanephroi isolated from transgenic ER-E2F1 suicide-inducible mice. E2F1 is a transcription factor that regulates cell proliferation and the ectopic expression of which induces apoptosis. The xenotissue components could therefore be cleared by apoptosis, leaving the autologous Epo-producing tissues [84]. Xenometanephroi* per se* could thus acquire some renal functions in the host omentum, as well as supplying a niche for host stem cells to regenerate renal tissues that can be rebuilt using host-cell components. These techniques may help to reduce the adverse effects of long-term immunosuppressant administration and to address the ethical issues surrounding xenotransplantation [85, 86].

## 8. Use of a Nephrogenic Niche for Growing Xenoembryos 

**Figure 2:**
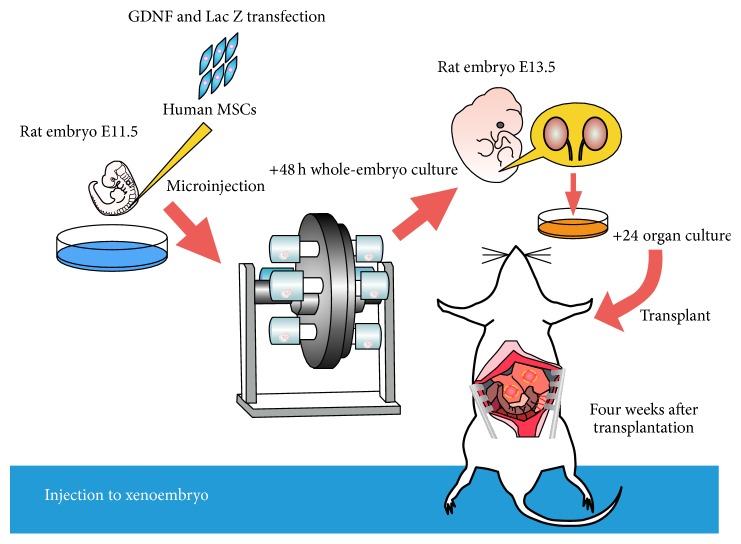
Strategy for nephron regeneration. MSCs were injected at the site of sprouting of the ureteric bud in E11.5 rat embryos. These embryos were then developed in whole-embryo culture for 48 h. Development of a neokidney derived from hMSCs in rat embryos. A kidney anlagen derived from MSCs was transplanted into the adult rat omentum. After culturing, hMSCs formed a neokidney in the rat omentum.

We exploited the developing xenoembryo as a niche for organogenesis using stem cells of renal lineage. Using this strategy, we previously showed that the xenobiotic developmental process for growing xenoembryos allows exogenous human MSCs to undergo epithelial conversion and form a nephron that produces urine and Epo [87–89] (Figure 2). During development of the metanephros, the MM initially forms from the caudal portion of the nephrogenic cord [90] and secretes GDNF, which induces the nearby Wolffian duct to produce a UB [91]. We generated a metanephros in organ culture by microinjecting GDNF-expressing transfected hMSCs into the site of budding, and the recipient embryo was grown in a whole-embryo culture system. Viral-free manipulation was performed using a thermoreversible GDNF polymer [92]. Donor hMSCs were integrated into the rudimentary metanephros and differentiated morphologically into tubular epithelial cells, interstitial cells, and glomerular epithelial cells [88]. We then transplanted the developed metanephros into the omentum to allow vascular integration from the recipient to form a functional nephron. As a result, an hMSC-derived “neokidney” was generated, which contained a human nephron associated with host vasculature [88]. This neokidney produced urine with higher concentrations of urea nitrogen and creatinine than in the sera of the recipient. This suggests that the neokidney, developed in the omentum, produced urine by hemofiltration [88]. Furthermore, the hMSC-derived neokidney secreted human Epo in response to the induction of anemia in the host animal [93]. This “organ factory” was thus able to preserve normal hormonal regulation to maintain Epo levels. However, the current system was unable to reconstruct a ureter or collecting tubules derived from the UB. To determine if MSCs could differentiate into a UB progenitor using chick embryos, hMSCs expressing PAX2 were injected into the chicken UB progenitor region [90]. The cells migrated caudally with the elongating Wolffian duct, integrated into the Wolffian duct epithelia, and expressed LIM1, demonstrating the ability of hMSCs to differentiate into Wolffian duct cells under the influence of local xenosignals [89]. These results indicate that it might be possible to reconstruct a whole kidney by transplanting renal lineage stem cells at a suitable time and location to regenerate derivatives of the MM and UB. Renal lineage stem cells as MSCs are easy to obtain in large numbers and are relatively cheap to establish. MSCs can be harvested from the patient as adult stem cells. We therefore used hMSCs as a cell source for kidney regeneration and succeeded in differentiating them into a nephron structure by GDNF-transfection and injection into a xenoembryo. Further studies are needed to verify the use of other adult kidney stem cells [42] or nephron progenitor cells [21] induced from iPSCs. With a view to the future clinical application of kidney regeneration, we also validated the effect of exposure of adult stem cells to uremic toxins over long periods and noted some differences in gene expression of stemness markers in MSCs from patients with ESKD. These results suggest that these cells may not be a suitable source for kidney regeneration [94].

Based on our previous promising results, we are currently investigating the possibility of large-scale harvesting of metanephroi from pigs, given that the volume of the kidney in pigs is almost identical to that in humans [92]. The ultimate size of the developed metanephros appears to be imprinted during the early stages of development in the host embryo. Another study also demonstrated that the body size and weight of interspecific chimeras (metanephroi derived from xenoembryos) conformed to the recipient species [34]. We hope that, by overcoming these challenges, this strategy might provide a novel direction for generating donor kidneys with a suitable size and function for transplantation.

## 9. Conclusion

This review has summarized recent research involving the use of renal stem cells and renal bioengineering to regenerate functional whole kidneys* de novo.* Despite significant recent advances, the reconstruction of a complete functional kidney remains difficult, and many problems are still unsolved. Direct differentiation of ESCs/iPSCs into nephron progenitors has not yet succeeded in generating mature functioning tissues* in vivo*. Before regenerated kidneys can be used in clinical practice, a method of generating fully functioning renal tissues that produce urine and Epo needs to be developed. Additionally, the regenerated tissue must be able to survive and function in the long term. Future research in stem cell biology and bioengineering will hopefully resolve these issues and open the door to new therapeutic strategies for kidney regeneration aimed at repairing kidney damage and restoring function.
